# Prenatal and early life stress and risk of eating disorders in adolescent girls and young women

**DOI:** 10.1007/s00787-016-0848-z

**Published:** 2016-04-15

**Authors:** Xiujuan Su, Hong Liang, Wei Yuan, Jørn Olsen, Sven Cnattingius, Jiong Li

**Affiliations:** 1 Department of Women’s and Children’s Health Care, Shanghai First Maternity and Infant Hospital, Tongji University School of Medicine, Shanghai, 200120 People’s Republic of China; 2 Key Laboratory of Reproduction Regulation of NPFPC, SIPPR, IRD, Fudan University, Shanghai, 200032 People’s Republic of China; 3 Department of Clinical Epidemiology, Aarhus University Hospital, Aarhus, 8200 N Denmark; 4 Clinical Epidemiology Unit, Department of Medicine, Karolinska University Hospital and Karolinska Institute, Stockholm, Sweden

**Keywords:** Bereavement, Eating disorders, Cohort study, Prenatal stress, Postnatal stress

## Abstract

Females are more likely than males to develop eating disorders (EDs) in the adolescence and youth, and the etiology remains unclear. We aimed to estimate the effect of severe early life stress following bereavement, the death of a close relative, on the risk of EDs among females aged 10–26 years. This population-based cohort study included girls born in Denmark (from 1973 to 2000) or Sweden (from 1970 to 1997). Girls were categorized as exposed if they were born to mothers who lost a close relative 1 year prior to or during pregnancy or if the girl herself lost a parent or a sibling within the first 10 years of life. All other girls were included in unexposed group. An ED case was defined by a diagnosis of EDs at ages of 10–26 years, including broadly defined bulimia nervosa, broadly defined anorexia nervosa and mixed EDs. Poisson regression models were used to estimate the incidence rate ratio (IRR) between exposed group and unexposed group.A total of 64453 (3.05 %) girls were included in the exposed group. We identified 9477 girls with a diagnosis of EDs, of whom 307 (3.24 %) were from the exposed group. Both prenatal and postnatal exposure following bereavement by unexpected death was associated with an increased overall risk of EDs (IRR_prenatal_: 1.49, 95 % CI: 1.01–2.19 and IRR_postnatal_: 1.34, 95 % CI: 1.05–1.71). We observed similar results for subtypes of broadly defined bulimia nervosa (IRR: 2.47, 95 % CI: 1.67–3.65) and mixed EDs (IRR: 1.45, 95 % CI: 1.02–2.07).Our findings suggest that prenatal and early postnatal life stress due to unexpected death of a close relative is associated with an increased overall risk of eating disorders in adolescent girls and young women. The increased risk might be driven mainly by differences in broadly defined bulimia nervosa and mixed eating disorders, but not broadly defined anorexia nervosa.

## Introduction

The prevalence of eating disorders (EDs) has been increasing over the past decades [1, 2]. EDs affect up to 3 % of population, and the prevalence varies by subtypes and populations [3, 4]. The cumulative risk of EDs among females is more than 17-fold higher than among males in Denmark [5]. EDs are among the psychiatric disorders with the highest mortality risks, comparable with schizophrenia and bipolar disorder in adolescents and young adults [6]. In addition, individuals with EDs are likely to suffer from other psychiatric diseases, such as autism spectrum disorders [7, 8]. However, the etiology of EDs is still poorly understood.

Animal studies have suggested that perinatal stress has negative effects on the development of hypothalamus pituitary adrenal (HPA) axis that plays an important role in regulating eating behaviors. Some have suggested that acute maternal stress has a greater long-term effect on HPA axis function and behavior than chronic maternal stress with sex-specific effects [9]. Findings from a series of animal experiments showed that a single aversive stimulus could change sensitivity of HPA axis on response to future stressors [10]. A cross-sectional study in humans observed that war stress could modify the eating behavior and was associated with an increased risk of EDs [11]. Another case–control study suggested that chronic stress and psychiatric comorbidity are strongly associated with the onset of EDs in adolescents, and psychiatric comorbidity is a partial mediating factor in the association of stress with EDs [12]. It is difficult to compare findings in these studies due to variations in assessment of stress and case diagnosis.

Prenatal stress following maternal bereavement due to death of a close relative, has been proposed to be related to several psychiatric diseases in the offspring, such as attention deficit hyperactivity disorder (ADHD), schizophrenia, autism spectrum disorders and suicide attempt in different timing patterns [13–17]. Specifically, severe stress to a mother during the first trimester may alter the risk of schizophrenia in offspring [13], while bereavement during preconception period is associated with an increasing risk of ADHD [14]. Our previous study has shown that bereavement related to loss of an older child or spouse other than other relatives is associated with an increased risk of EDs in infants and toddlers [18]. In addition, it has been suggested that there are still certain specific risk factors for each subtype of EDs except sharing the same risk factors [19]. Hence, we hypothesized that maternal bereavement might be associated with risk of EDs in the offspring with different timing pattern for each subtype of EDs. Using data from national registers in Denmark and Sweden, we aimed to examine the association between prenatal and early postnatal life stress due to bereavement and risk of EDs in adolescent girls and young women. We expected that the association differed by timing of exposure [17], and type of death [18], and the subtype of EDs [19].

## Methods

We performed a population-based cohort study based on several national registries from Denmark and Sweden. The study was approved by the Danish Data Protection Agency (No. 2008-41-2680) and the local ethics committee in Central Region of Denmark (No. M-201000252) and Karolinska Institutet (No. 2008/4:6).

### Data sources

A unique personal identification number in Scandinavian countries allows individual records linkage across these national registries. The international classification of diseases (ICD) criteria was used during the study period, Specifically, eighth version (ICD-8) from 1977 to 1993 in Denmark and from 1973 to 1986 in Sweden, and ICD-9 from 1987 to1996 in Sweden, and the ICD-10 from 1994 onwards in Denmark and from 1997 onwards in Sweden.

### Participants and follow up

We used data from the Danish Civil Registry System and the Swedish Multi-generation Register to identify girls born in Denmark from 1970 to 2000 (*N* = 1,034,539) and girls born in Sweden from 1973 to 1997 (*N* = 1,246,560). We excluded girls with no linkage to the mothers (*N* = 3672), with missing or implausible maternal age (unknown or ≤13 or ≥60 years, *N* = 3579), and born to mothers who lost a close relative by death due to EDs (*N* = 67). Girls who died (*N* = 16,029) or emigrated (*N* = 59,416) or were diagnosed with EDs (*N* = 4418) before the start of the follow-up period were also excluded. Although DSM-5 no longer requires amenorrhea for a diagnosis of AN, it has been reported that puberty has a close association with onset of EDs and age of menarche has declined, the start of follow up time was set to 10 years of age. To ensure that exposure precedes the onset of the disease, girls exposed to bereavement between 10–26 years of age were excluded (*N* = 83,162). Our study population consisted of 2,110,756 girls born in Denmark (*N* = 934,610) and Sweden (*N* = 1,178,146).

All subjects were followed from 10 years of age until the first diagnoses of EDs, death, emigration, the day when they reach 26 years of age, or end of follow-up (December 31, 2010 for Denmark and December 31, 2007 for Sweden), whichever came first.

### Exposure

We categorized the girls as exposed if they were either prenatally or postnatally exposed to stress following bereavement by death of a family member. The prenatal exposure referred to maternal bereavement due to death of an older child, spouse, or one of other family members (a sibling or a parent) in the prenatal period from 1 year before or during pregnancy to the birth of the girl. The postnatal exposure referred to bereavement when the girl lost a mother, father or a sibling during the period from birth to up to 10 years of age. If the girl experienced more than once the exposure (i.e.,death) during the above defined time window, we gave priority to the earliest exposure.

We subdivided the exposure according to: (1) relationship to the deceased relative: death by a core family member (father and sibling for prenatal exposure, mother, father, and sibling for postnatal exposure) and death by an extended family member (mother’s parent or mother’s sibling, only suitable for prenatal exposure); (2) timing of exposure: prenatal exposure was further divided into five time periods (12–7 months before pregnancy, 6–0 months before pregnancy, the first trimester, the second trimester and the third trimester); postnatal exposure were divided into three time periods (infants and toddlers: 1–2 years old, preschoolers: 3–5 years old, and school age: 6–10 years old) [20]; (3) type of death: unexpected death [codes were for Sweden: 79590–79599, 79621, E807-E999 (ICD-8); 798, E807-E999 (ICD-9); R95, R96, R98, V01-Y98 (ICD-10); and for Denmark: 795, 810–823, 950–959, 800–807, 825–949, 960–999 (ICD-8); R95-R98, V01-V89, X60-X84 (ICD-10)] and other death.

### Outcome

Information on EDs was obtained from the Danish Psychiatric Central Research Register, Danish National Patient Register, and Swedish Patient Register. We categorized EDs into three subtypes according to the following ICD codes: (1) broadly defined anorexia nervosa, including feeding disturbance (ICD-8 codes 306.5), anorexia nervosa (ICD-9 codes 307.B and ICD-10 codes F50.0), and atypical anorexia nervosa (ICD-10 codes F50.1); (2) broadly defined bulimia nervosa, including bulimia nervosa (ICD-9 codes 307.F and ICD-10 codes F50.2), atypical bulimia nervosa (ICD-10 codes F50.3); and (3) mixed eating disorders, including overeating associated with other psychological disturbances (ICD-10 codes F50.4), vomiting associated with other psychological disturbances (ICD-10 codes F50.5), other eating disorders (ICD-10 codes F50.8), and eating disorder unspecified (ICD-10 codes F50.9). Patients with more than one type of EDs were categorized according to the first diagnosis.

### Statistical analysis

All data management and analyses were performed with the SAS version 9.2 statistical software packages (SAS Institute, Inc., Cary, North Carolina). Because survival analysis (Cox’s proportional hazards) would often be too computationally intensive for a dataset of this size with time-dependent variables, Log-Linear Poisson regression models, as an approximation of the Cox’s regression model [21], were used to estimate the incidence rate ratio (IRR) of EDs in relation to the exposure.

The following variables were adjusted for in the analyses: country (Denmark, Sweden), birth order (1st, 2nd, ≥3rd, missing), the number of fetuses in the current pregnancy (1, >1, missing), maternal education (<9 years, 10–14 years, ≥15 years, missing), calendar age of the index girl, calendar year of follow up, and family history of psychiatric diseases (yes, no) which was defined by the fact that father, mother or sibling of the index girl was diagnosed with psychiatric diseases (ICD-8 codes 290–315, ICD-9 codes 290–319 and ICD-10 codes F00–F99). Maternal age and paternal age were also adjusted as continuous variables in the analyses. We also restricted analyses to girls without family history of psychiatric diseases to partly disentangle the genetic effects. We first presented results for all exposure categories, and then presented results according to the timing of exposure (prenatal exposure and postnatal exposure).

## Results

A total of 64,453 (3.05 %) girls were exposed during 1 year before pregnancy through 10 years of age. The baseline characteristics of the study population are presented in Table 1. Exposed girls were more likely to be born to older parents, or to have a family history of psychiatric diseases, or to have a higher birth order.

**Table 1:** Baseline characteristic of population at birth by bereavement exposure

	Exposed (*N* = 64,453)	Unexposed (*N* = 2,046,302)
Country		
Denmark	25,921 (40.22)	908,689 (44.41)
Sweden	38,532 (59.78)	1,137,614 (55.59)
The number of fetuses in current pregnancy		
Singleton	59,693 (92.61)	1,852,949 (90.55)
Multiple	1888 (2.93)	44,534 (2.18)
Missing	2872 (4.46)	148,820 (7.27)
Birth order		
1st	24,279 (37.67)	915,401 (44.73)
2nd	21,816 (33.85)	684,946 (33.47)
≥3rd	15,874 (24.63)	346,635 (16.94)
Missing	2484 (3.85)	99,321 (4.85)
Maternal age (years)		
<27	24,086 (37.37)	889,427 (43.47)
27–30	17,403(27.00)	597,789 (29.21)
>30	22,964(35.63)	559,087 (27.32)
Paternal age (years)		
<29	21,203 (32.90)	807,665 (39.47)
29–33	19,649 (30.49)	678,635 (33.16)
>33	23,042 (35.75)	530,648 (25.93)
Missing	559 (0.87)	29,355 (1.43)
Family history of psychiatric diseases		
Yes	17,333 (26.89)	379,973 (18.57)
No	47,120 (73.11)	1,666,330 (81.43)
Maternal education (years)		
<10	16,446 (25.52)	444,753 (21.73)
10–14	30,182(46.83)	1,012,346 (49.47)
>14	7745 (12.02)	265,688 (12.98)
Missing	10,080 (15.64)	323,516 (15.81)

We identified 9477 females with a diagnosis of EDs, of whom 307 (3.24 %) were exposed to either prenatal or postnatal bereavement (Table 2). We did not find an overall association between exposure to bereavement and the risk of EDs (IRR: 1.06, 95 % confidence interval (CI): 0.94–1.19). When the exposure was categorized by type of death, we found that exposure to bereavement caused by unexpected death was associated with an increased overall risk of EDs (IRR: 1.38, 95 % CI: 1.12–1.69), both for prenatal (IRR: 1.49, 95 % CI: 1.01–2.19) and postnatal (IRR: 1.34, 95 % CI: 1.05–1.71) exposure. No association was observed after categorizing the exposure according to the timing of exposure and relationship of the deceased relative with the index girl.

**Table 2:** Incidence rate ratio (IRR) for eating disorders after exposure to bereavement, by timing of bereavement, relationship to the deceased, and type of death

Exposure	No. of cases	IR (10,000 person years)	Crude IRR	Adjusted IRR^a^
Non-exposure	9170	4.16	1.00	1.00
Any time exposure	307	4.45	1.07 (0.95–1.19)	1.06 (0.94–1.19)
Timing of exposure				
Prenatal exposure	150	4.66	1.11 (0.95–1.31)	1.06 (0.90–1.25)
12–7 months	47	5.13	1.22 (0.92–1.62)	1.15 (0.86–1.54)
6–0 months	53	5.03	1.20 (0.91–1.57)	1.16 (0.89–1.53)
During pregnancy	50	4.00	0.97 (0.74–1.28)	0.91 (0.69–1.21)
1st trimester	13	3.62	0.86 (0.50–1.48)	0.85 (0.49–1.46)
2nd trimester	21	4.11	1.02 (0.67–1.55)	1.01 (0.66–1.53)
3rd trimester	16	4.21	1.00 (0.61–1.64)	0.86 (0.51–1.44)
Postnatal exposure	157	4.28	1.02(0.87–1.20)	1.05(0.86–1.24)
0–2 years	44	5.03	1.20 (0.89–1.61)	1.29 (0.95–1.77)
3–5 years	45	4.03	0.96 (0.72–1.29)	0.96 (0.70–1.31)
6–10 years	68	4.05	0.98 (0.77–1.24)	0.98 (0.46–1.27)
Relationship to the deceased				
Loss of a core relative	188	4.35	1.04 (0.90–1.20)	1.05 (0.90–1.23)
Prenatal maternal loss of child/spouse	31	4.77	1.13 (0.80–1.61)	1.09 (0.76–1.56)
Postnatal loss of a parent	100	4.16	1.00 (0.82–1.22)	1.03 (0.83–1.27)
Postnatal loss of a sibling	57	4.50	1.07 (0.82–1.39)	1.08 (0.82–1.43)
Loss of other relatives	119	4.63	1.11 (0.93–1.33)	1.06 (0.88–1.27)
Type of death				
Any time of unexpected death	99	5.62	1.35 (1.11–1.64)	1.38 (1.12–1.69)
Prenatal unexpected death	27	6.60	1.57 (1.08–2.29)	1.49 (1.01–2.19)
Postnatal unexpected death	72	5.32	1.28 (1.02–1.62)	1.34 (1.05–1.71)
Any time of other death	208	4.05	0.97 (0.84–1.11)	0.95 (0.82–1.90)
Prenatal other death	123	4.39	1.05 (0.88–1.26)	1.01 (0.84–1.20)
Postnatal other death	85	3.67	0.87 (0.70–1.08)	0.87 (0.69–1.10)

*IR* incidence rate, *IRR* incidence rate ratio

^a^Adjustment for country, birth order, the number of fetuses in current pregnancy, paternal age, maternal age, maternal education, calendar age of the index girl, calendar year of follow up, and family history of psychiatric diseases

We did not observe any significant association between bereavement and the risk of broadly defined anorexia nervosa (IRR: 0.96, 95 % CI: 0.82–1.13) (Table 3). Similar to the overall risk of EDs, we observed exposure to bereavement caused by unexpected death in prenatal or postnatal period was associated with the increased risks of broadly defined bulimia nervosa (IRR: 2.47, 95 % CI: 1.67–3.65) (Table 4) and mixed EDs (IRR: 1.45, 95 % CI: 1.02–2.07) (Table 5). However, the significant association disappeared for mixed EDs when separating the time window of exposure into prenatal (IRR: 1.81, 95 % CI: 0.97–3.37) and postnatal exposure (IRR: 1.33, 95 % CI: 0.86–2.04). We did not observe that the associations for all three subtypes differed by the timing of exposure and relationship of the deceased with the subject.

**Table 3:** Incidence rate ratio (IRR) for broadly defined anorexia nervosa after exposure to bereavement, by timing of bereavement, relationship to the deceased, and type of death

Exposure	No. of cases	IR (10,000 person years)	Crude IRR	Adjusted IRR^a^
Non-exposure	5712	2.59	1.00	1.00
Any time exposure	166	2.41	0.96 (0.82–1.12)	0.96 (0.82–1.13)
Timing of exposure				
Prenatal exposure	81	2.52	1.02 (0.82–1.27)	0.96 (0.77–1.20)
12–7 months	26	2.84	1.12 (0.75–1.67)	1.07 (0.72–1.58)
6–0 months	28	2.66	1.09 (0.75–1.58)	1.06 (0.73–1.54)
During pregnancy	27	2.16	0.88 (0.61–1.29)	0.79 (0.53–1.17)
1st trimester	4	1.11	0.45 (0.17–1.23)	0.44 (0.17–1.17)
2nd trimester	14	2.74	1.12 (0.66–1.89)	1.09 (0.64–1.84)
3rd trimester	9	2.37	0.97 (0.50–1.87)	0.73 (0.35–1.52)
Postnatal exposure	85	2.32	0.90 (0.73–1.12)	0.97 (0.76–1.22)
0–2 years	24	2.74	1.08 (0.71–1.62)	1.13 (0.72–1.77)
3–5 years	27	2.42	0.92 (0.62–1.36)	0.90 (0.58–1.40)
6–10 years	34	2.02	0.80 (0.57–1.13)	0.93 (0.65–1.32)
Relationship to the deceased				
Loss of a core relative	96	2.22	0.87 (0.71–1.07)	0.93 (0.74–1.16)
Prenatal maternal loss of child/spouse	11	1.69	0.69 (0.38–1.25)	0.74 (0.41–1.34)
Postnatal loss of a parent	54	2.25	0.85 (0.64–1.12)	0.96 (0.72–1.29)
Postnatal loss of a sibling	31	2.45	1.00 (0.70–1.43)	0.97 (0.65–1.44)
Loss of other relative	70	2.72	1.10 (0.87–1.39)	1.01 (0.79–1.28)
Type of death				
Any time of unexpected death	43	2.44	0.98 (0.72–1.32)	1.05 (0.76–1.44)
Prenatal unexpected death	9	2.20	0.90 (0.47–1.73)	0.81 (0.41–1.63)
Postnatal unexpected death	34	2.51	1.00 (0.71–1.41)	1.13 (0.79–1.62)
Any time of other death	123	2.40	0.95 (0.79–1.14)	0.94 (0.77–1.14)
Prenatal other death	72	2.57	1.04 (0.82–1.31)	0.98 (0.78–1.24)
Postnatal other death	51	2.20	0.85 (0.64–1.13)	0.87 (0.64–1.19)

*IR* incidence rate, *IRR* incidence rate ratio

^a^Adjustment for country, birth order, the number of fetuses in current pregnancy, paternal age, maternal age, maternal education, calendar age of the index girl, calendar year of follow up, and family history of psychiatric diseases

**Table 4:** Incidence rate ratio (IRR) for broadly defined bulimia nervosa after exposure to bereavement, by timing of bereavement, relationship to the deceased, and type of death

Exposure	No. of cases	IR (10,000 person years)	Crude IRR	Adjusted IRR^a^
Non-exposure	1659	0.75	1.00	1.00
Any time exposure	63	0.97	1.27 (0.96–1.67)	1.30 (0.97–1.72)
Timing of exposure				
Prenatal exposure	25	0.78	1.08 (0.71–1.69)	1.23 (0.80–1.89)
12–7 months	8	0.87	1.10 (0.49–2.46)	1.21 (0.54–2.71)
6–0 months	7	0.66	0.96 (0.43–2.13)	1.02 (0.46–2.28)
During pregnancy	10	0.80	1.21 (0.63–2.33)	1.43 (0.74–2.76)
1st trimester	3	0.83	1.40 (0.45–4.35)	1.63 (0.55–5.26)
2nd trimester	4	0.78	1.31 (0.49–3.51)	1.58 (0.59–4.23)
3rd trimester	3	0.79	0.89 (0.22–3.55)	1.00 (0.25–4.01)
Postnatal exposure	38	1.03	1.42 (0.99–2.03)	1.35 (0.93–1.96)
0–2 years	10	1.14	1.35 (0.64–2.83)	1.41 (0.66–2.99)
3–5 years	7	0.63	0.90 (0.41–2.02)	0.92 (0.41–2.05)
6–10 years	21	1.25	1.80 (1.13–2.87)	1.60 (0.98–2.63)
Relationship to the deceased				
Loss of a core relative	47	1.09	1.48 (1.08–2.04)	1.38 (0.98–1.92)
Prenatal maternal loss of child/spouse	9	1.38	1.81 (0.86–3.81)	1.48 (0.70–3.12)
Postnatal loss of a parent	27	1.12	1.61 (1.07–2.43)	1.48 (0.95–2.29)
Postnatal loss of a sibling	11	0.87	1.06 (0.53–2.13)	1.11 (0.55–2.22)
Loss of other relative	16	0.62	0.92 (0.54–1.55)	1.13 (0.97–1.92)
Type of death				
Any time of unexpected death	30	1.70	2.67 (1.84–3.89)	2.47 (1.67–3.65)
Prenatal unexpected death	9	2.20	3.70 (1.92–7.13)	3.87 (2.01–7.47)
Postnatal unexpected death	21	1.55	2.36(1.50–3.72)	2.07 (1.28–3.34)
Any time of other death	33	0.64	0.79 (0.53–1.18)	0.86 (0.57–1.28)
Prenatal other death	16	0.57	0.72 (0.41–1.27)	0.82 (0.46–1.44)
Postnatal other death	17	0.73	0.87 (0.49–1.54)	0.91 (0.51–1.61)

*IR* incidence rate, *IRR* incidence rate ratio

^a^Adjustment for country, birth order, the number of fetuses in current pregnancy, paternal age, maternal age, maternal education, calendar age of the index girl, calendar year of follow up, and family history of psychiatric diseases

**Table 5:** Incidence rate ratio (IRR) for mixed eating disorders, following exposure to bereavement, by timing of bereavement, relationship to the deceased, and type of death

Exposure	No. of cases	IR (10,000 person years)	Crude IRR	Adjusted IRR^a^
Non-exposure	3049	1.38	1.00	1.00
Any time exposure	110	1.60	1.19 (0.97–1.45)	1.11 (0.90–1.36)
Timing of exposure				
Prenatal exposure	55	1.71	1.34 (1.02–1.76)	1.19 (0.90–1.57)
12–7 months	19	2.07	1.54 (0.95–2.47)	1.32 (0.81–2.16)
6–0 months	20	1.90	1.49 (0.95–2.34)	1.37 (0.87–2.15)
During pregnancy	16	1.28	1.06 (0.65–1.73)	0.93 (0.56–1.54)
1st trimester	6	1.67	1.38 (0.62–3.07)	1.29 (0.58–2.88)
2nd trimester	4	0.78	0.65 (0.24–1.73)	0.60 (0.23–1.60)
3rd trimester	6	1.58	1.31 (0.59–2.91)	1.02 (0.42–2.45)
Postnatal exposure	55	1.50	1.06 (0.79–1.41)	1.03 (0.76–1.39)
0–2 years	16	1.83	1.32 (0.78–2.34)	1.48 (0.88–2.51)
3–5 years	18	1.61	1.04 (0.61–1.76)	1.01 (0.61–1.80)
6–10 years	21	1.25	0.94 (0.60–1.47)	0.79 (0.47–1.31)
Relationship to the deceased				
Loss of a core relative	71	1.64	1.17 (0.91–1.51)	1.11 (0.85–1.46)
Prenatal maternal loss of child/spouse	16	2.46	1.78 (1.06–3.02)	1.53 (0.88–2.64)
Postnatal loss of a parent	37	1.54	1.00 (0.69–1.44)	0.91 (0.61–1.36)
Postnatal loss of a sibling	18	1.42	1.18 (0.74–1.87)	1.24 (0.78–1.97)
Loss of other relative	39	1.52	1.22 (0.89–1.68)	1.10 (0.79–1.52)
Type of death				
Any time of unexpected death	35	1.99	1.50 (1.06–2.13)	1.45 (1.02–2.07)
Prenatal unexpected death	11	2.69	2.03 (1.09–3.77)	1.81 (0.97–3.37)
Postnatal unexpected death	24	1.77	1.35 (0.86–2.05)	1.33 (0.86–2.04)
Any time of other death	75	1.46	1.08 (0.85–1.38)	0.99 (0.77–1.27)
Prenatal other death	44	1.57	1.24 (0.91–1.68)	1.10 (0.80–1.50)
Postnatal other death	31	1.34	0.89 (0.60–1.32)	0.84 (0.54–1.29)

*IR* incidence rate, *IRR* incidence rate ratio

^a^Adjustment for country, birth order, the number of fetuses in current pregnancy, paternal age, maternal age, maternal education, calendar age of the index girl, calendar year of follow up, and family history of psychiatric diseases

We observed similar results when analyses were restricted to subjects without family history of psychiatric diseases (results are available upon request).

## Discussion

Using nationwide registers from two Nordic countries, we examined associations between stress due to bereavement from 1 year preconception to 10 years of age and the risks of EDs, including its three subtypes among adolescent girls and young women. We observed an increased risk of EDs among girls who were exposed to either prenatal or early postnatal life stress due to unexpected death of a close relative. It seemed that the increased risk was driven by differences in broadly defined bulimia nervosa and mixed EDs rather than broadly defined anorexia nervosa. Timing does not seem to be important.

Dysfunction of HPA axis has been implicated in the pathogenesis of EDs [22], and not only prenatal stress but also postnatal stress could alter HPA axis function [23, 24]. Girls exposed to both prenatal and postnatal stress are reported to have an increased vulnerability to psychiatric diseases and decreased stress response possibly mediated through a process of increased corticotrophin-releasing hormone in the hippocampus which regulates HPA axis activities [25]. This is also partly consistent with the results that functional signs of neonatal dysmaturity had a significant additive interaction with childhood abuse in determining the risk for the illness [26]. Stress due to loss of a close relative occurred in the early prenatal period could persist in the following period, even in postnatal period. Hence, our results that both prenatal and postnatal bereavement were associated with an increased risk of EDs might be contributed by a combined influence of prenatal maternal stress on fetal neurodevelopment and self-response to stress in early childhood. Our results also have alternative explanation that severe stress reduced maternal food intake and subsequently influenced the intrauterine nutrition [27, 28].

An animal study has suggested that effect of stress on food intake is more dependent on the intensity of a stressor [29]. Normally, sudden or violent death causes more stress than other types of death [30]. Cute maternal stress (mostly unexpected stress) has a greater long-term effect on HPA function and behavior than chronic maternal stress. These findings are in line with our results on association between unexpected loss of a close relative and increased risk of EDs.

To our knowledge, this is the first large-scale study to examine the association between prenatal and early life stress and the risk of EDs with its subtypes. Patients with different types of EDs show different personality traits and social adaption capacities [31]. EDs subtypes during adolescence are different in term of neuro correlates of inhibitory control and activation of brain regions [32]. We found an increased risk for broadly defined bulimia nervosa among girls who experienced unexpected loss of a close relative both during prenatal period and within 10 years after birth. The potential specific mechanisms need to be further studied.

We did not find an increased risk of broadly defined anorexia nervosa in bereaved girls. Higher education level and ascending year of birth have been proposed as risk factors for anorexia nervosa [33, 34]. We observed the same pattern for broadly defined anorexia nervosa in the present study. An increased risk of mixed EDs was observed among girls after exposure to bereavement caused by unexpected death. However, this association was weaker than the corresponding association for broadly defined bulimia nervosa and was not significant after dividing it into prenatal and postnatal exposure. The number of cases with mixed EDs was probably limited by time and ICD version: more than 30 % patient was diagnosed mixed EDs based on the ICD-10 in this study. Hence, it is worthy to further explore the associations between stress and subtypes of EDs using a more detailed categorized system of EDs. Further studies are also needed to clarify the possible mechanisms involve in different subtypes of EDs.

This study has several strengths. We used a large cohort with prospectively collected information that included all the girls born in Denmark and Sweden during nearly two decades. This allowed us to explore the association between rare exposure (bereavement) and outcomes (EDs and its subtypes), and to adjust for a number of potential confounders. In this population-based cohort study, bias due to selection of population and loss of follow up is unlikely. In addition, bereavement due to loss of a close relative, widely accepted as a relatively precise and universal indicator for stress, was used as an indicator of exposure status [35]. Again, this is the first large-scale study to examine the association between bereavement and different patterns for subtypes.

Our study also has several limitations. First, the ICD system was used to identify the cases in this study, instead of the DSM-5 that has more detailed diagnosis information about the EDs [36]. Some misclassification of EDs may be expected. Specifically, the incidence rate of EDs could be underestimated because cases who did not present for treatment would not be included at all. However, this needs not to be related to exposure, and hence would bias the estimates toward the null. Second, we did not find any validation study of EDs in the three registries. The Swedish Patient Register may have a high predictive value for most validated diagnoses (85-95 %), but a low sensitivity [37]. Validity of some psychiatric diseases is high in Danish Psychiatric Central Research Register, like childhood autism (94 %) and schizophrenia (90 %) [38, 39]. In our study, broadly defined anorexia nervosa was the most common subtype of EDs, which is in line with the previous studies [2, 5]. Third, we only have inpatients before 1995 in Denmark and all time in Sweden. Bereavement might either lower or increase the threshold for hospitalization, therefore comorbidity or other factors related to the threshold of hospitalization should be considered in the future studies. Fourth, as an observational study based on register data, it is not possible to conclude that the association is causal and further studies are needed.

In conclusion, our findings suggested that prenatal and early postnatal life stress due to unexpected death of a close relative is associated with an increased overall risk of eating disorders in adolescent girls and young women. The increased risk might be driven mainly by differences in broadly defined bulimia nervosa and mixed eating disorders, but not broadly defined anorexia nervosa.

## References

[1] Darby A, Hay P, Mond J, Quirk F, Buttner P, Kennedy L (2009). The rising prevalence of comorbid obesity and eating disorder behaviors from 1995 to 2005. Int J Eat Disord.

[2] Hoek HW (2006). Incidence, prevalence and mortality of anorexia nervosa and other eating disorders. Curr Opin Psychiatr.

[3] Neumark-Sztainer D, Hannan PJ (2000). Weight-related behaviors among adolescent girls and boys—results from a national survey. Arch Pediatr Adolesc Med.

[4] Treasure J, Claudino AM, Zucker N (2010). Eat disord. Lancet.

[5] Pedersen CB, Mors O, Bertelsen A, Waltoft BL, Agerbo E, McGrath JJ, Mortensen PB, Eaton WW (2014). A comprehensive nationwide study of the incidence rate and lifetime risk for treated mental disorders. JAMA Psychiatry.

[6] Hoang U, Goldacre M, James A (2014). Mortality following hospital discharge with a diagnosis of eating disorder: national record linkage study, England, 2001-2009. Int J eat disord.

[7] Berkman ND, Lohr KN, Bulik CM (2007). Outcomes of eating disorders: a systematic review of the literature. Int J eat disord.

[8] Swinbourne J, Hunt C, Abbott M, Russell J, St Clare T, Touyz S (2012). The comorbidity between eating disorders and anxiety disorders: prevalence in an eating disorder sample and anxiety disorder sample. Aust N.Z J Psychiatry.

[9] Emack J, Matthews SG (2011). Effects of chronic maternal stress on hypothalamo-pituitary-adrenal (HPA) function and behavior: no reversal by environmental enrichment. Horm Behav.

[10] Marti O, Garcia A, Velles A, Harbuz MS, Armario A (2001). Evidence that a single exposure to aversive stimuli triggers long-lasting effects in the hypothalamus-pituitary-adrenal axis that consolidate with time. Eur J Neurosci.

[11] Aoun A, Garcia FD, Mounzer C, Hlais S, Grigioni S, Honein K, Dechelotte P (2013). War stress may be another risk factor for eating disorders in civilians: a study in Lebanese University Students. Gen Hosp Psychiat.

[12] Rojo L, Conesa L, Bermudez O, Livianos L (2006). Influence of stress in the onset of eating disorders: data from a two-stage epidemiologic controlled study. Psychosom Med.

[13] Khashan AS, Abel KM, McNamee R, Pedersen MG, Webb RT, Baker PN, Kenny LC, Mortensen PB (2008). Higher risk of offspring schizophrenia following antenatal maternal exposure to severe adverse life events. Arch Gen Psychiatry.

[14] Li J, Olsen J, Vestergaard M, Obel C (2010). Attention-deficit/hyperactivity disorder in the offspring following prenatal maternal bereavement: a nationwide follow-up study in Denmark. Eur Child Adolesc Psychiatry.

[15] Class QA, Abel KM, Khashan AS, Rickert ME, Dalman C, Larsson H, Hultman CM, Langstrom N, Lichtenstein P, D’Onofrio BM (2014). Offspring psychopathology following preconception, prenatal and postnatal maternal bereavement stress. Psychol Med.

[16] Walder DJ, Faraone SV, Glatt SJ, Tsuang MT, Seidman LJ (2014). Genetic liability, prenatal health, stress and family environment: risk factors in the Harvard Adolescent Family High Risk for Schizophrenia Study. Schizophr Res.

[17] St-Hilaire A, Steiger H, Liu A, Laplante DP, Thaler L, Magill T, King S (2015). A prospective study of effects of prenatal maternal stress on later eating-disorder manifestations in affected offspring: preliminary indications based on the Project Ice Storm cohort. Int J Eat Disord.

[18] Su X, Xu B, Liang H, Olsen J, Yuan W, Cnattingius S, Laszlo KD, Li J (2015). Prenatal maternal bereavement and risk of eating disorders in infants and toddlers: a population-based cohort study. BMC Psychiatry.

[19] Jacobi C, Hayward C, de Zwaan M, Kraemer HC, Agras WS (2004). Coming to terms with risk factors for eating disorders: application of risk terminology and suggestions for a general taxonomy. Psychol Bull.

[20] Canada PHAo: http://www.phac-aspc.gc.ca/publicat/mh-sm/divorce/4-eng.php. Accessed 15 Jan 2005

[21] Andersen P, Borgan Ø, Gill R, Keiding N (1995). Statistical models based on counting processes.

[22] Putignano P, Dubini A, Toja P, Invitti C, Bonfanti S, Redaelli G, Zappulli D, Cavagnini F (2001). Salivary cortisol measurement in normal-weight, obese and anorexic women: comparison with plasma cortisol. Eur J. endocrinol Eur Federation Endocr Soc.

[23] Ladd CO, Huot RL, Thrivikraman KV, Nemeroff CB, Plotsky PM (2004). Long-term adaptations in glucocorticoid receptor and mineralocorticoid receptor mRNA and negative feedback on the hypothalamo-pituitary-adrenal axis following neonatal maternal separation. Biol Psychiatry.

[24] O’Donnell KJ, Glover V, Barker ED, O’Connor TG (2014). The persisting effect of maternal mood in pregnancy on childhood psychopathology. Dev Psychopathol.

[25] Lupien SJ, McEwen BS, Gunnar MR, Heim C (2009). Effects of stress throughout the lifespan on the brain, behaviour and cognition. Nat Rev Neurosci.

[26] Favaro A, Tenconi E, Santonastaso P (2010). The interaction between perinatal factors and childhood abuse in the risk of developing anorexia nervosa. Psychol Med.

[27] Krebs H, Macht M, Weyers P, Weijers HG, Janke W (1996). Effects of stressful noise on eating and non-eating behavior in rats. Appetite.

[28] Torres SJ, Nowson CA (2007). Relationship between stress, eating behavior, and obesity. Nutrition.

[29] Marti O, Marti J, Armario A (1994). Effects of chronic stress on food intake in rats: influence of stressor intensity and duration of daily exposure. Physiol Behav.

[30] Kaltman S, Bonanno GA (2003). Trauma and bereavement: examining the impact of sudden and violent deaths. J Anxiety Disord.

[31] Ahren-Moonga J, Holmgren S, von Knorring L (2008). Af Klinteberg B: Personality traits and self-injurious behaviour in patients with eating disorders. Eur Eat Disord Rev J Eat Disord.Associ.

[32] Lock J, Garrett A, Beenhakker J, Reiss AL (2011). Aberrant brain activation during a response inhibition task in adolescent eating disorder subtypes. Am J Psychiatry.

[33] Ahren-Moonga J, Silverwood R, Klinteberg BA, Koupil I (2009). Association of higher parental and grandparental education and higher school grades with risk of hospitalization for eating disorders in females: the Uppsala birth cohort multigenerational study. Am J Epidemiol.

[34] Steinhausen HC, Jakobsen H, Helenius D, Munk-Jorgensen P, Strober M (2014). A nation-wide study of the family aggregation and risk factors in anorexia nervosa over three generations. The International journal of eating disorders.

[35] Pfeffer CR, Altemus M, Heo M, Jiang H (2007). Salivary cortisol and psychopathology in children bereaved by the September 11, 2001 terror attacks. Biol Psychiatry.

[36] Fairburn CG, Cooper Z (2011). Eating disorders, DSM-5 and clinical reality. Br J Psychiatry J Ment Sci.

[37] Ludvigsson JF, Andersson E, Ekbom A, Feychting M, Kim JL, Reuterwall C, Heurgren M, Olausson PO (2011). External review and validation of the Swedish national inpatient register. BMC Public Health.

[38] Uggerby P, Ostergaard SD, Roge R, Correll CU, Nielsen J (2013). The validity of the schizophrenia diagnosis in the Danish Psychiatric Central Research Register is good. Dan Med J.

[39] Lauritsen MB, Jorgensen M, Madsen KM, Lemcke S, Toft S, Grove J, Schendel DE, Thorsen P (2010). Validity of childhood autism in the Danish Psychiatric Central Register: findings from a cohort sample born 1990-1999. J Autism Dev Disord.

